# Deformity Reconstruction Surgery for Tibial Hemimelia

**DOI:** 10.3390/children8060461

**Published:** 2021-05-31

**Authors:** David Y. Chong, Dror Paley

**Affiliations:** 1 Department of Orthopedic Surgery and Rehabilitation, University of Oklahoma Health Sciences Center, Oklahoma City, OK 73104, USA; 2 Paley Orthopedic & Spine Institute at St. Mary’s Medical Center, West Palm Beach, FL 33407, USA

**Keywords:** tibial hemimelia, tibial deficiency, absence of tibia, tibial aplasia, Paley classification, treatment, patelloplasty, fibula centralization

## Abstract

Tibial hemimelia is a rare congenital deficiency with a wide spectrum of pathology and deformity. This paper aims to give a comprehensive review of tibial hemimelia, with a concise summary of the history, pathology, and clinical findings of tibial hemimelia, while providing treatment recommendations and a review of the current literature. Classifications and surgical treatments are discussed, including amputation, limb reconstruction, and lengthening. Type-specific treatments are also discussed, including staged distraction correction of joint contractures of knee and ankle, Weber patelloplasty, fibular centralization, knee and ankle arthrodesis, implantable articulated distractors, and the role of femoral shortening. Amputation is a simpler and easier solution for many patients; however, reconstruction options continue to evolve, improve, and provide better functional outcomes in many cases. Factors favoring surgical reconstruction include the presence of a knee joint/proximal tibia, and the presence of a patella and quadriceps mechanism.

## 1. Introduction

Tibial hemimelia is extremely rare, with a reported incidence of approximately one in a million live births [1,2]. It was first mistakenly reported in 1841 [3] and correctly reported by Billroth in 1861 [4,5]. In 1941, there were 79 published cases [6], and since then there have been several hundred more reported. Though the presentation can be variable, tibial hemimelia commonly presents as a shortened leg with knee and ankle deformity. The tibia may be hypoplastic, completely absent, or a non-ossified remnant (anlage) that is invisible on radiographs. Knee flexion contractures are common, and there may be instability from missing collateral ligaments, and the patella and quadriceps extensor mechanism may be absent. Dimples may be present in the skin over the knee joint. The fibula may be dysplastic, and it is often subluxated or dislocated either proximally or distally. The ankle often presents in varus and equinus with an adducted and supinated foot. The medial side of the foot may have a hypoplastic big toe or be missing rays. Duplication of toes, metatarsals, tarsals, fibulas, and femurs are also characteristic.

## 2. Evaluation

Initial evaluation should include a full set of radiographs. The presence, absence or partial presence of the tibia will help guide treatment. However, in younger children, non-ossified cartilage or an anlage will not be visible. A well-developed distal femur may suggest but does not guarantee the presence of a proximal tibia [7]. More information can be obtained from serial radiographs as the child matures, but magnetic resonance imaging (MRI) and ultrasound may be utilized to confirm further detail, especially if no proximal tibia is present on radiographs.

Dissection of specimens with complete tibia aplasia has revealed more deficiencies within the anterior and deep posterior compartments. The posterior tibial bundle is present but shortened, and anomalous tendons may tether the foot in supination. A skin dimple is commonly found over the proximal fibula or over the knee if the patella is missing. Subtalar coalitions were common, and the talus was found to articulate with the distal medal fibula. Most had toe anomalies, ranging from four to eight digits [8,9].

Tibial hemimelia may be diagnosed with prenatal ultrasound by 16 weeks of gestation [10]. Tibial hemimelia is bilateral in 30% of cases [11]. Unilateral cases seem affect the right side more often [12]. The genetic inheritance of tibial hemimelia varies. Reports have described parent to child transmission [6,13] and families with multiple affected siblings [14,15]. Autosomal dominant and autosomal recessive inheritance has been described [16,17,18,19,20]. Consanguinity has also been implicated [21]. It has been postulated that the pathology is due to a mesoblast origin, as opposed to a mechanical or traumatic source [22,23]. It may also have variable phenotypic manifestations, demonstrated by a report of identical twins with only one twin affected [24].

## 3. Associations

Tibial hemimelia is associated with Werner’s syndrome [25,26], Langer–Giedion syndrome [27], Gollop–Wolfgang complex [28], and CHARGE syndrome [29,30,31,32]. About 60% of cases have associated anomalies [33,34]. Ipsilateral deformities at the thigh and knee include congenital femoral deficiency, a missing patella or quadriceps extensor mechanism, knee hyperextension or flexion, and a bifid femur. Foot deformities include clubfeet, syndactyly, missing or duplicated toes, diplopodia, ectrodactyly, micromelia, and a mirror foot deformity [35,36,37,38,39,40,41,42,43]. Other associated deformities include coxa valga, hip dysplasia or dislocation, radial dysplasia, lobster claw deformity, hand syndactyly and polydactyly, triphalagism, mirror hand, missing fingers or toes, hemivertebrae, and myelomeningocele [44,45,46,47]. In addition, cleft palate, deafness, cryptorchidism, pseudo-hermaphroditism, and hypospadias have also been associated with tibial hemimelia [14].

Due to the varied presentation of the tibia, other disorders may be easily confused for tibial hemimelia. Fibular hemimelia may present with a congenitally shortened tibia and fibula, or there could be complete absence of the fibula. With fibular hemimelia, the ankle and foot are often in valgus. In tibia hemimelia, they are usually in a varus position. Tibial hemimelia should also be distinguished from a congenital knee deficiency that is associated with thrombocytopenia-absent-radius syndrome. 

## 4. Classification

**Figure 1:**
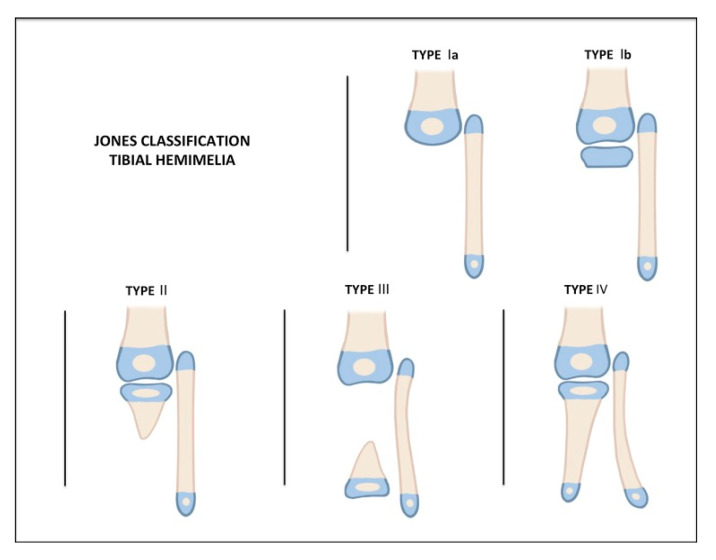
Jones classification of tibial hemimelia.

Beyond the basic classification of congenital deficiencies described by Frantz and O’Rahilly [48], the Jones classification (Figure 1) in 1978 has been commonly used [13]. This scheme is based on radiographs. Type I deficiencies do not have a tibia that is visible on radiographs. The Ia group has a distal femoral epiphysis that is hypoplastic, whereas the Ib group has normal ossification that suggests that the proximal tibial epiphysis is still present. Type II deficiencies have a visible tibia proximally, but it is deficient distally. Type III have a visible distal tibia, but they are deficient proximally. Finally, the Type IV deficiencies are marked by distal tibiofibular diastasis along with tibial shortening. Birch proposed adding a type V to include limbs with tibial shortening but with an intact proximal and distal epiphysis [49].

The Weber classification takes into the account the cartilaginous anlage, if present, and has seven types and 12 subtypes, which includes a few rarer forms of tibial hemimelia that did not fit into the Jones classification [50,51]. However, this can become a cumbersome classification to use. 

**Figure 2:**
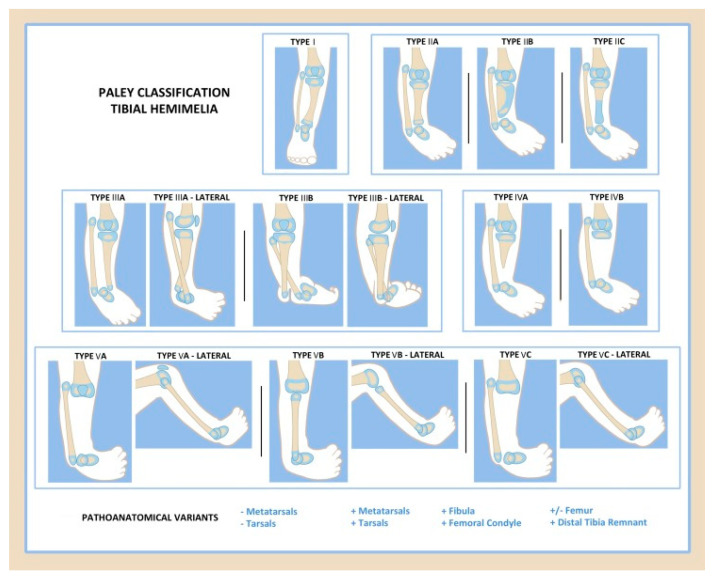
Paley classification of tibial hemimelia.

The Paley classification was originally proposed in 2003 and modified in 2015 [52,53,54]. This classification is oriented around a progression of deficiency from least to most severe. There are five main types and 11 subtypes (Figure 2 and Table 1). Type 1 is a hypoplastic but nondeficient tibia with relative overgrowth of the proximal fibula. Type 2 has a proximal and distal tibia epiphysis but a dysplastic ankle. Subtypes include: (A) well-formed distal tibia physis, (B) a delta tibia or bracket epiphysis, and (C) delayed ossification or a cartilaginous anlage, with a missing distal tibial physis. Type 3 has distal tibio-fibula diastasis and is missing the distal tibia plafond, but the proximal tibia is well formed. Type 3A often has the talus located between the tibia and fibula due to the lack of tibial plafond. Type 3B has a skin cleft of varying depths separating the tibia and fibula, with the foot attached to the fibular side. Type 4 is marked by distal tibial aplasia with preservation of the proximal tibial epiphysis. Type 4A has a proximal tibial physis and metaphysis that is ossified, and the deficiency starts at the level of the diaphysis. Type 4B has delayed ossification of the proximal tibial epiphysis with no physis present. Type 5 is complete tibial aplasia with a knee flexion contracture. Type 5A has both an intact patella and intact quadriceps function and an equinovarus contracture of the foot. Type 5B has no patella but has an intact quadriceps, and an auto-centralized fibula. Type 5C has no patella or distal quadriceps. Plus and minus modifiers can also be added for the duplication or deficiency of toes, metatarsals, tarsals, fibula, distal tibial remnant, femoral condyle, or femur.

Prior to the Paley classification, the wide spectrum of pathology of tibial hemimelia could not fit into any classification scheme, as demonstrated by new case reports every year [55,56,57]. The Paley classification allows for inclusion of the entire spectrum of deficiencies and duplications. When comparing the Jones, Weber, and Paley classifications, the latter was the only one that was able to classify all types of tibial hemimelia in a series of 113 cases [54]. It is also the only classification that guides treatment and prognosis. Each type and subtype have a separate reconstructive algorithm, which is described in the following section. 

## 5. Treatment Options

Early reconstructive treatments for complete absence of the tibia have included fibular centralization, with fusion or arthroplasty [58,59]. For partial absence, early reports describe synostosing the tibial remnant to the fibula [45,60]. For an intercalary defect, the contralateral fibula has been transposed with success [61]. Other reported operations have included fusion of the fibula and talus, transfer of the proximal fibula to the intercondylar notch, and side-to-side synostosis of the fibula and tibia proximally and distally [46,62].

For most surgeons, the simplest and easiest treatment may be amputation. In complete tibial absence (Jones type Ia/Paley 5), most studies lean towards amputation [33,47,63,64]. This may not be acceptable to some patients or cultures. In a single-center study cohort in India, only one patient out of 24 opted for amputation, despite the severity of the deformity, and the authors noted that cultural acceptance of amputation in India is low [65]. Reconstructive limb salvage options are available, though more severe deformity may require more complex surgery [12,66,67,68]. The presence of an active quadriceps mechanism and a tibial anlage allows for better reconstructive options and prevention of knee flexion deformities [69]. A recent retrospective cohort study found that reconstructed limbs had better functional outcomes than amputation [70]. Advanced imaging such as MRI and ultrasound may be useful to help determine the presence of the patella, proximal tibial anlage, and quadriceps muscles in the younger patient, and thus help determine a treatment strategy [71]. 

### 5.1. Reconstruction

Fibular transfer and centralization was first published and developed by Brown in 1965 [72]. This procedure was performed on patients with complete tibial aplasia, concurrently with a Syme-type amputation of the foot. Forty-five percent of patients required a secondary surgery due to a knee flexion deformity, and most wore braces for ambulation. Patients without quadriceps function had inferior results [73]. Most authors have reported poor outcomes with the Brown procedure, in which many reconstructive efforts went on to knee disarticulation [49,64,74,75]. Poor outcomes were attributed to knee instability, poor range of motion, and progressive knee flexion contractures. More recent case series have had slightly better results, but most still ambulate with a brace with limited knee range of motion [76,77,78,79]. Again, the presence of a strong quadriceps mechanism, a patella, and a proximal tibia or anlage favor reconstruction, and tibiofibular synostosis has generally met with good results. 

Modern reconstruction efforts have included the use of circular external fixators for soft tissue distraction [53,54,80,81]. Laufer et al., in their series of 12 limbs and 10 patients with complete tibia aplasia, used an external fixator for soft-tissue distraction followed by a staged reconstruction. Overall, soft tissue distraction with external fixators was successful in preparing for a second stage surgery. However, their results were not successful by the standards of Jayakumar and Eilert [74], who defined a good result as achieving adequate gait with no flexion contracture, varus or valgus instability of <5°, and a minimum active range of motion of 10°–80°. No patients achieved a range of motion of 10°–80°, but all achieved coronal stability, except those treated with a Weber patelloplasty, and 50% of patients had secondary reconstructive procedures, including a repeat external frame distraction to re-centralize the fibula. However, at final follow-up, all patients were now ambulatory with a knee-ankle-foot orthosis (KAFO) and able to participate in activities of daily living. All families felt that surgery provided major improvements, and no patients have had a secondary amputation. Thus, the expected outcomes of reconstruction must continue to be tempered for complete tibial aplasia: the patient will be improved from baseline and ambulatory with orthotics, but persistent contractures, instability, and recurrence are still a real threat.

With the presence of a proximal tibia (Jones type II, Paley type 4a) or a tibial anlage (Jones type Ib, Paley type 4b), tibiofibular synostosis has shown good results, with only mild residual knee flexion contractures and otherwise stable knee joints [63,65,79]. Many did have distal amputations at the ankle and use a prosthesis. They have had good stability and mild knee flexion contractures. For Jones type Ib and II, tibiofibular synostosis is recommended [33].

Distal tibia aplasia in Jones type II deficiencies leads to ankle instability, and can either be treated with arthrodesis or amputation. Calcaneo-fibular fusions with Boyd amputations, Syme, and Chopart amputations have been described [33,39,63,82]. In cases with distal tibia and fibula diastasis, a distal synostosis and ankle fusion has been described if an external fixator is not available [83].

The Weber patelloplasty describes a complex procedure, in which the patella is converted into a tibial plateau [84,85]. The fibula is centralized and fused to the patella, and the knee flexion contracture is gradually corrected with an external fixator. Paley has published a modification of this technique [54]. His modification includes soft tissue distraction of the fibula from the femur and talus from the fibula, to centralize the fibula under the femur and the talus under the fibula using an external fixator. Then, the patella is fused to the head of the fibula and BMP is used if the patella is unossified. There is still a paucity of published results of this technique, though Weber did further describe and refine his procedure in 2006. Both Paley and Weber report achieving a mobile, stable knee joint with active and passive knee range of motion. Laufer reported on two patients treated with a Weber patelloplasty after external fixator distraction, with 2- and 8-year follow-up [80]. Both had significant contractures and coronal instability necessitating KAFO usage, but were ambulatory and still improved from baseline.

Both the surgeon and the family must be aware of the guarded prognosis of reconstruction, especially in the most severe deficiencies. Unfortunately, the long-term results of reconstruction are still sparse in the literature. Short term results and expert opinion can certainly give some guidance, but they must be guarded, given the deterioration of results due to recurrence, dislocation, and instability. Hopefully, future studies from major reconstructive centers will help our understanding of the prognosis and outcomes of surgical reconstruction of the more severe forms of tibial hemimelia. 

### 5.2. Amputation

Knee disarticulation has been previously described for treatment and it remains as a primary salvage option for failed Brown procedures. If the femur is severely deficient, a femoro-fibular arthrodesis may be performed to effectively lengthen the femur for improved prosthetic fitting [63]. In Schoenecker’s series, 86% of deficient limbs eventually had some type of an amputation [33]. He recommended knee disarticulation unless a proximal tibia or anlage is present. Some authors support early amputation, as the patient would treat it more like a congenital amputation and quickly adapt to their prosthesis and rehabilitation [39]. In patients with significant knee instability, one study has found significantly improved outcome measures and recommends amputation over reconstruction [86]. For some patients and families, they may prefer a quicker, more definitive solution, as opposed to months or even years of reconstructive surgery. 

If going down the amputation route, most surgeons will opt for a through-knee amputation for Jones type 1 (Paley type 5), a through or below-knee amputation for Jones type 2 (Paley type 4a), and a Syme’s amputation for Jones type 4 (Paley type 3a). With modern prosthetics, amputation leads to good functional results and is likely the most reliable and predictable method of treatment. However, amputation can also have complications, which in this specific patient population may include prosthetic irritation from the prominent fibular head and progressive varus deformity [12]. 

### 5.3. Limb Lengthening

Limb lengthening is commonly needed at least as an adjunct to reconstructive options due to the significant shortening of the tibia and fibula [51,87]. The leg length discrepancy in tibial hemimelia typically remains proportional over time; thus, the final predicted leg length discrepancy can be calculated to help families make educated decisions [88,89]. However, it is interesting to note that one case report described significant femoral overgrowth of 6 cm after reconstruction without lengthening, which the authors attribute to stimulation of axial-directed stresses of short-leg ambulation [90]. Staged lengthenings allow trained surgeons to achieve 5–8 cm of lengthening using external fixation. Bone formation can be delayed due to the smaller diameter and cortical nature of the fibula that is substituting for a tibia [91]. 

## 6. Author’s Type-Specific Reconstruction

Due to the rarity and wide spectrum of tibial hemimelia, treatment continues to evolve and change. Most of the current literature consists of small case series or expert opinion, and long-term results are still sparse. As a disclaimer, the following section is mostly based on the expert opinion of the senior author, who has experience with more than 250 tibial hemimelia reconstructions as an alternative to amputation. The senior author’s current reconstruction strategies are summarized and depicted as follows.

### 6.1. Paley Type 1 

These patients tend to have bilateral involvement and may be familial and autosomal dominant. The tibia is shortened relative to the femur, creating a mesomelic disproportion and short stature, and proximal valgus deformity is often present. Temporary hemiepiphysiodesis of the proximal medial tibia is used to correct the valgus deformity, and may be combined with epiphysiodesis of the proximal fibula which tends to overgrow. In unilateral cases, lengthening with deformity correction will equalize the limb length discrepancy. If the discrepancy is small, contralateral epiphysiodesis can be considered. In bilateral cases, only the valgus deformity may need to be corrected, though bilateral lengthening for correction of disproportion and short stature may be considered. 

### 6.2. Paley Type 2 

**Figure 3:**
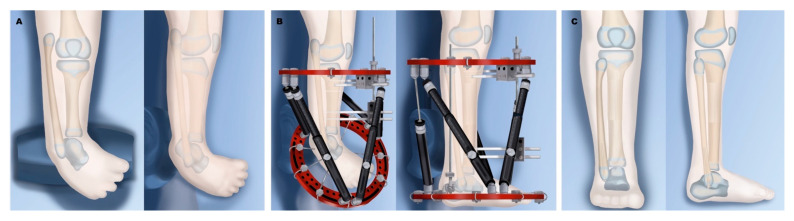
Treatment of Paley type 2A. (**A**) Typical deformity with shortened tibia and equinovarus foot and overgrown proximal fibula. (**B**) Application of external fixator for staged correction of foot equinovarus, distal fibular transport, and finally lengthening of the tibia. (**C**) Results after tibial lengthening with distal fibular screw epiphysiodesis.

**Figure 4:**
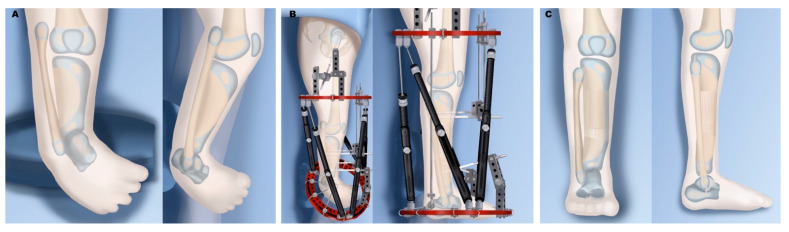
Treatment of Paley type 2B. (**A**) Bracket epiphysis deformity of tibia. (**B**) Application of external fixator after excision of bracket, with staged gradual foot correction, distal fibular transport, and tibial osteotomy for lengthening. (**C**) Results after completion. The foot has been corrected to a plantigrade position, the fibula is at station with a distal epiphysiodesis, and the tibia is longer due to lengthening.

**Figure 5:**
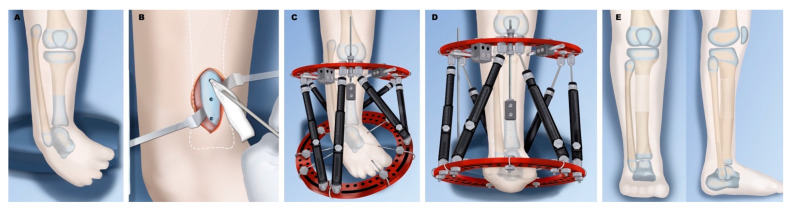
Treatment of Paley type 2C. (**A**) Distal tibia cartilaginous anlage with no physis. (**B**) Insertion of bone morphogenic protein (BMP) into tibial anlage. (**C**) External fixator for correction of foot deformity and distal fibular transport. (**D**) Tibial osteotomy and lengthening with fixator. (**E**) Final results after tibial lengthening with fibula at station and foot plantigrade.

In type 2A (Figure 3), the foot is found in marked equinovarus, and it is internally rotated relative to the knee. Using an external fixator, the foot can be gradually distracted and brought back over to a reduced position, followed by a tibial osteotomy and lengthening to match the fibula length. In type 2B (Figure 4), the bracket epiphysis is resected, and a tibial osteotomy can also be done for acute or gradual correction. For acute correction, a fibular shortening osteotomy is needed to correct the varus deformity. This is followed by gradual lengthening at a separate time. For gradual correction, the external fixator is applied to the femur and extends down to attach to the upper tibia. Gradual angular correction and lengthening are then performed. In type 2C (Figure 5), there is delayed ossification of the distal tibial anlage, which can be confirmed on MRI. The treatment is similar to that of type 2A, with the addition of bone morphogenic protein (BMP) insertion into the tibial anlage when the lengthening osteotomy is performed. The use of BMP in children is still considered off-label use, and it may cause localized swelling but have had few directly attributable complications [92]. 

### 6.3. Paley Type 3

**Figure 6:**
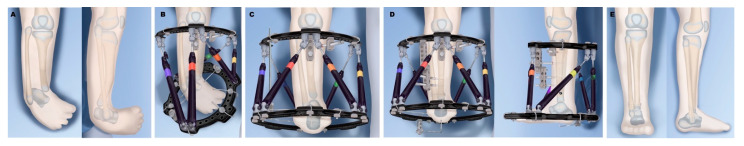
Treatment of Paley type 3A. (**A**) Distal tibia-fibula diastasis. The tibial plafond is absent and the end of the tibia is what normally would have been a medial malleolus; foot and fibula internally rotated around tibia. (**B**) Application of external fixator for gradual correction of foot and fibula position. (**C**) Foot centralized under end of tibia, ready for tibiotalar arthroplasty. (**D**) Distal tibia reshaping to talus and stabilization of distal tibia and fibula diastasis and osteotomy of tibia for diaphyseal straightening. (**E**) Final result after hardware removal with plantigrade foot and distal fibula screw epiphysiodesis.

**Figure 7:**
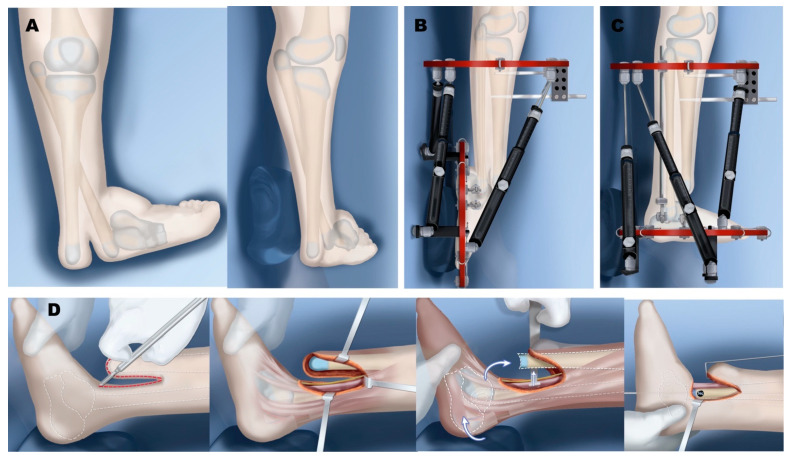
Treatment of Paley type 3B. (**A**) Distal tibia and fibula diastasis with skin cleft. Fibula is associated with talus and foot. (**B**) Application of external fixator for distraction and gradual correction. (**C**) Foot now in plantigrade position. (**D**) Excision and closure of skin cleft performed at time of diastasis stabilization and tibiotalar biologic arthroplasty as in type 3A.

The type 3A deformity pattern (Figure 6) typically presents with the foot internally rotated around the tibia. An external fixator is used to gradually distract and externally rotate the foot with the fibula, relative to the tibia. The foot is then corrected out of equinovarus into a plantigrade position, with the talus under the tibia. A second stage surgery is then performed, reshaping the joint surface of the tibia to the talus and closing the tibia and fibula diastasis. The diastasis is stabilized with a syndesmotic suture and washer system. The type 3B deformity pattern (Figure 7) appears much worse, but the treatment is similar. During the second surgery, the skin cleft between the distal tibia and fibula is closed. 

### 6.4. Paley Type 4

**Figure 8:**
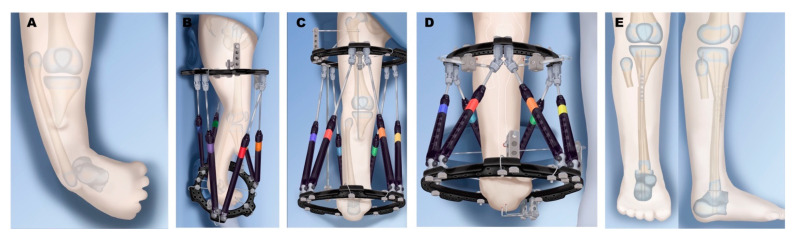
Treatment of Paley type 4A. (**A**) Well-formed proximal tibia and knee with distal tibia aplasia. (**B**) External fixator used to gradually correct equinovarus foot position and distally transport fibular head. (**C**) Fibula brought down to station and foot plantigrade. (**D**) Fibular osteotomy performed with transfer to proximal tibia. Distal fibula epiphysis is fused to talus without disrupting the physis. Fusion stabilized with intramedullary retrograde wires. (**E**) Final results after fixator removal.

In the Paley type 4, the knee joint is present and functional, but it may be missing cruciate ligaments. The amount of hypoplasia of the tibia varies. In type 4A (Figure 8), the proximal tibia is ossified, proximal tibial physis is present, and the knee joint functions normally. The fibula is transported distally and then transferred to the proximal tibia by means of an open dissection between the two bones and a fibular osteotomy. This author’s and other’s experiences have shown excellent results [93]. Though the knee remains functionally normal, the foot is in very severe equinovarus, and there is no distal tibia. Attempted ankle joint reconstruction and arthroplasty with the distal fibula has not been successful. At this time, the best option is to gradually distract the foot under the fibula. Subsequently, the talus is fused to the distal epiphysis in a physeal-sparing fashion, cutting only into the epiphysis and preserving the physis, stabilizing with an intramedullary wire. This is preferred to acute foot centralization by calcaneofibular arthrodesis [94]. For this author, the results of treatment of type 4A have been reliable and predictable with the methods described. 

**Figure 9:**
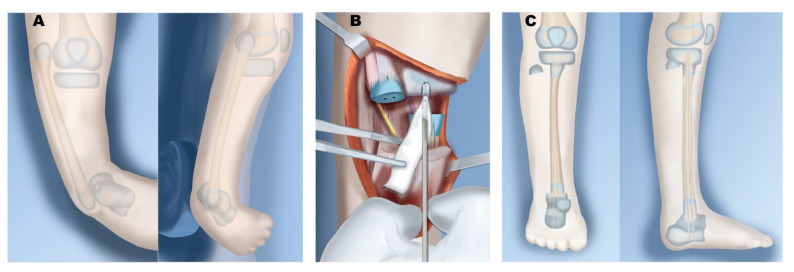
Treatment of Paley type 4B. (**A**) Unossified proximal tibial anlage with no physis. Initial treatment the same as 4A, with fibula distraction and foot correction. (**B**) Insertion of BMP into proximal tibia anlage. (**C**) Fixator removal following healing from physeal sparing proximal fibula osteotomy and transfer to proximal tibia. Physeal-sparing fusion of distal fibula to talus.

In type 4B (Figure 9), there is an unossified proximal tibial anlage. This tibial remnant provides knee stability and motion. All of these patients have a patella and a quadriceps mechanism. Thus, the treatment is the same as above for type 4A. The fibula is distracted from its proximally migrated position. The fibula is then osteotomized and transferred to the tibia. Additionally, BMP is inserted into the tibial anlage to help it ossify and promote union with the fibula. Since there is no physis, there will not be any proximal tibial growth. 

### 6.5. Paley Type 5

**Figure 10:**
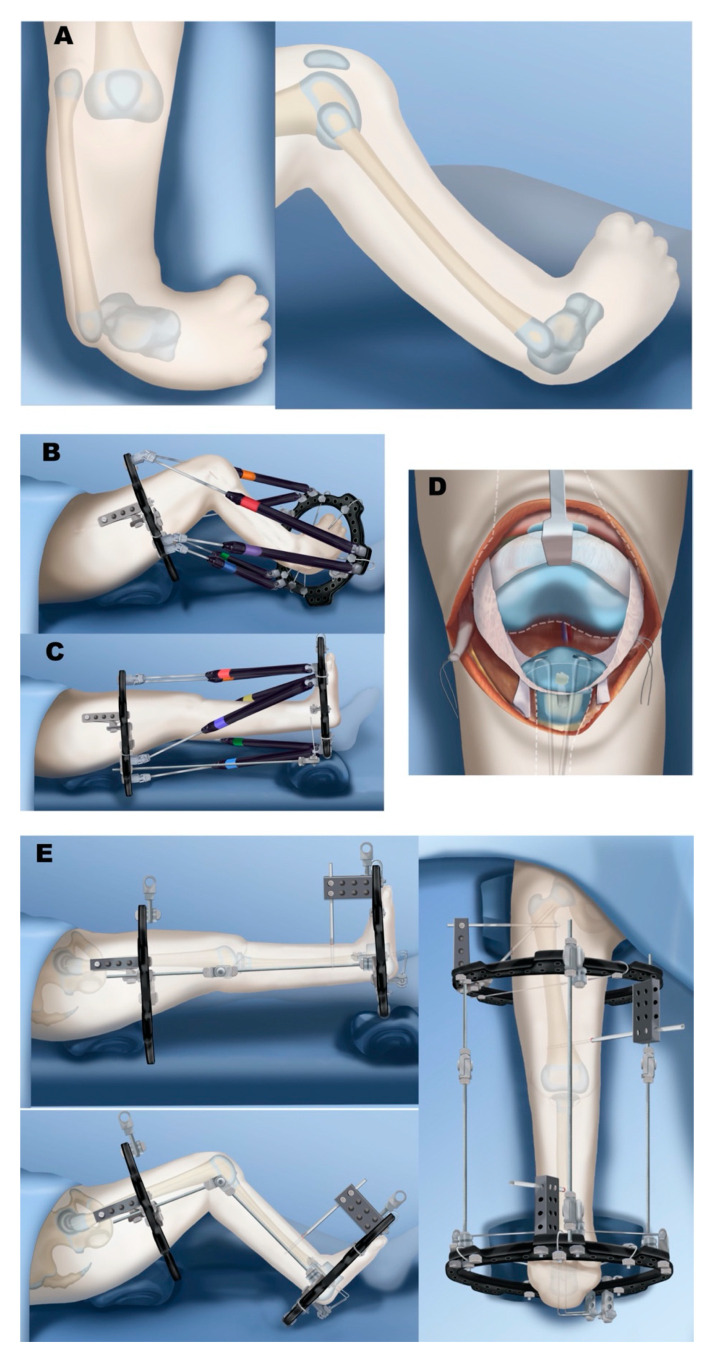
Treatment of Paley type 5A. (**A**) Complete aplasia of tibia, but patella and quadriceps are present. (**B**) External fixator placement for gradual fibula distraction and foot correction. (**C**) Knee flexion contracture corrected to straight position, preparing for physeal sparing patellar arthroplasty and physeal sparing talo-fibular fusion. (**D**) Paley-Weber patelloplasty converting the patella into a tibial plateau. (**E**) Hinged external fixator to protect arthroplasty but allow knee motion.

The Paley type 5 (Figure 10) is defined by a complete absence of the tibia. In addition, the patella and quadriceps tendon may not be present, and the biggest challenge is the absence of a knee joint. The ankle and foot can be treated as described for type 4. While knee fusion through femoro-fibulo-calcaneal arthrodesis has been described with satisfactory results [95], it is not the author’s preferred treatment approach, as it can be debilitating in bilateral cases [81]. Until recently, centralization of the fibula by modifications of the Brown procedure yielded unacceptable results, and amputation was preferred over these prior options. Recent advances that make reconstruction more tenable and appealing are the patellar arthroplasty concept developed by Weber and the femoral shortening approach developed by Paley. 

The Weber patellar arthroplasty is performed when the patella is present. The patellar arthroplasty fuses the patella to the head of the fibula. The patella is uniquely shaped to the distal femur in all positions of flexion and extension. By connecting the fibula to the patella, the patella acts like a tibial plateau. The author has modified the Weber procedure by first distracting the fibula into a centralized position followed by patellar arthroplasty. This can also be done in a physeal and nerve sparing fashion. Ideally, the final result is a knee that has active and passive motion from 0–90°, though this can still be difficult to achieve. This groundbreaking procedure has made saving and reconstructing the knee a practical reality, and the senior author (DP) has been performing this procedure since 2003. 

**Figure 11:**
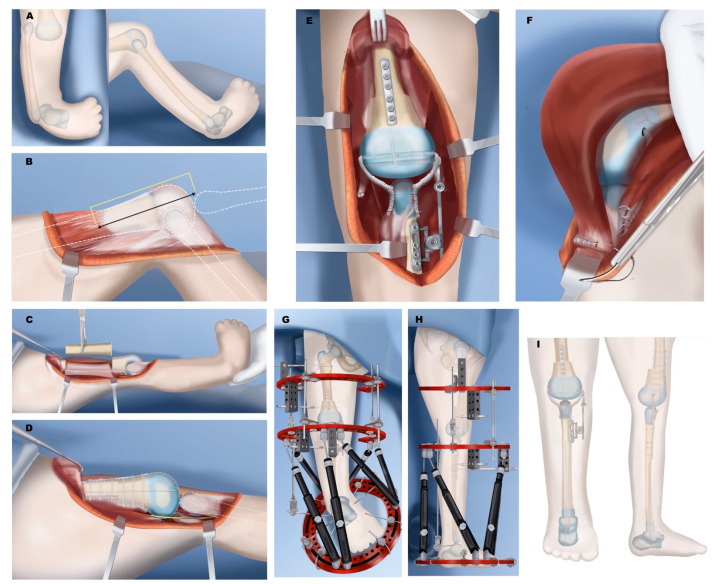
Treatment of Paley type 5C. (**A**) Complete aplasia of tibia with no patella. (**B**) Quadriceps are distally absent and end in distal femur. Femur needs to be shortened significantly to be able to bring the quadriceps to the level of the knee joint. (**C**) Femoral shortening osteotomy. (**D**) Plate fixation and intramedullary pinning of femoral osteotomy and distalization and centralization of fibula. (**E**) Knee stabilized with IJS internal articulated joint distractor. Collateral ligaments made from autograft or allograft. Reconstruction of collateral ligaments and placement of internal joint distraction system. (**F**) After quadricepsplasty, quadriceps muscle is advanced and sutured to the fibula head. (**G**) Placement of two-level external fixator: upper two rings with hinges for articulated stabilization of knee; lower two rings for gradual foot distraction. (**H**) Once talus is beneath the fibula and foot at 90°, physeal sparing fusion of foot to talus is carried out. (**I**) Final results after fixator removal. The IJS device side arm is disconnected 6 months later to allow for proximal fibular growth.

If the patella is not present, then reconstruction is a less attractive option. For severe types 5B and 5C (Figure 11), reconstruction currently consists of femoral shortening, quadricepsplasty, transfer of the quadriceps muscle to the fibular head, and application of an internal articulated joint distractor (IJS System, Skeletal Dynamics, Miami FL). This device is essentially an implantable internal hinge that allows flexion and extension of the knee while providing stability in the other planes. These advances have made reconstruction more feasible for the most severe type 5 cases, but long-term outcomes are still not known.

## 7. Conclusions

Both the rarity and spectrum of the presentation of tibial hemimelia make it a complex and difficult deformity to treat. Many early attempts at reconstruction have failed and converted to amputation, but surgical techniques have improved over time and can provide excellent outcomes in experienced hands. It is important to classify the type of tibial hemimelia in order to determine prognosis and develop a reconstructive plan. Partial deficiency of the tibia in Paley types 1 through 4 can be very successfully treated by reconstruction. However, for Paley type 5 deficiencies, through-knee amputation should be weighed against reconstruction, especially if the patella is not present. Reconstructive surgery for the treatment of tibial hemimelia has improved over the past decade and will continue to evolve, but long-term outcomes have not been reported for the complex reconstructions of complete tibial agenesis (Paley type 5). 

## Figures and Tables

**Table 1:** Paley classification of tibial hemimelia.

	Knee Joint	Proximal Tibia	Tibial Shaft	Distal Tibia	Ankle Joint/Foot
Type 1	Normal	Normal or valgus	Shortened	Normal	Normal
Type 2	Normal	Normal or mild dysplasia (pagoda shaped)	Shortened	Variable:	Dysplastic; equinovarus foot
2A				Well formed	
2B				Delta tibia	
2C				Cartilage anlage	
Type 3	Normal	Normal	Varus/procurvatum	No plafond	Diastasis; equinovarus
3A					Internally rotated around tibia
3B					Skin cleft, foot with fibula
Type 4	Normal	Present	Variable:	Absent	Equinovarus
4A		Normal	Partial		
4B		Non-ossified/dysplastic	Absent		
Type 5	Flexion contracture	Complete aplasia	Absent	Absent	Equinovarus
5A	(+)Quad (+)Patella				
5B	(+)Quad (−)Patella	Autocentralized fibula			
5C	(−)Quad (−)Patella	No knee capsule			

## Data Availability

Not applicable.
